# Supplementary material for: Using DNA metabarcoding and direct behavioural observations to identify the diet of proboscis monkeys (Nasalis larvatus) in the Kinabatangan Floodplain, Sabah
Source: PLoS One. 2025 Jan 3;20(1):e0316752. doi: 10.1371/journal.pone.0316752 (PMC11698349; doi:10.1371/journal.pone.0316752)
Supplement: S1 Fig — (DOCX) [file pone.0316752.s001.docx]

**SUPPORTING INFORMATION**


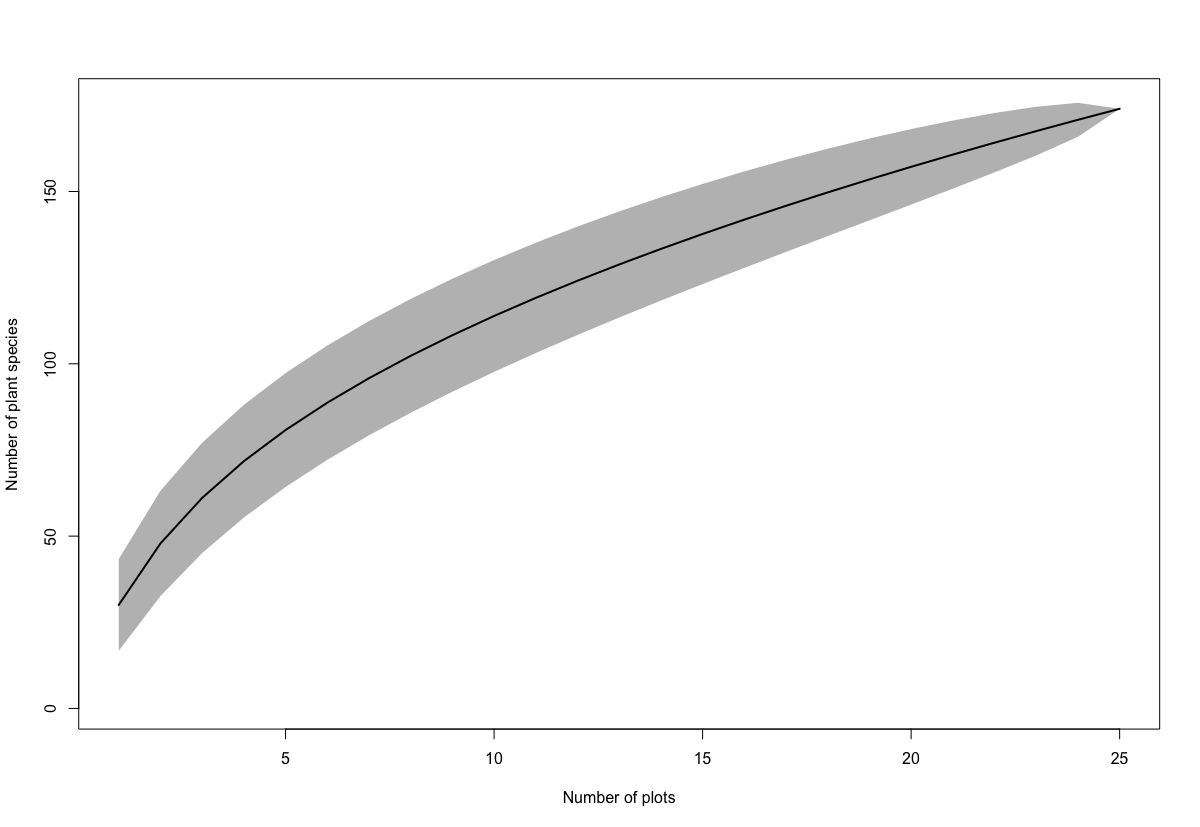


S1 Fig. Accumulation curve of plant species found in 25 botanical plots in Lot 6 of the LKWS
