# Supplementary material for: Using DNA metabarcoding and direct behavioural observations to identify the diet of proboscis monkeys (Nasalis larvatus) in the Kinabatangan Floodplain, Sabah
Source: PLoS One. 2025 Jan 3;20(1):e0316752. doi: 10.1371/journal.pone.0316752 (PMC11698349; doi:10.1371/journal.pone.0316752)
Supplement: S1 Table — (DOCX) [file pone.0316752.s002.docx]

**SUPPORTING INFORMATION**

S1 Table. List of plant taxa (n=67) consumed by proboscis monkeys at the riverside (recorded by instantaneous scan and *ad libitum* sampling observation methods, from May 2015 to March 2017)

| **Family** | **Species** | **Observation method sampling (n)** |
| --- | --- | --- |
| Anacardiaceae | *Buchanania arborescens* | *Ad libitum* |
|  | *Dracontomelon dao* | Scan (16) |
| Annonaceae | *Cananga odorata* | *Ad libitum* |
|  | *Polyalthia obliqua* | *Ad libitum* |
| Apocynaceae | *Alstonia sp.* | Scan (1) |
|  | *Alstonia iwahigensis* | *Ad libitum* |
|  | *Rauvolfia sumatrana* | *Ad libitum* |
| Araceae | *Pothos brevistylus* | *Ad libitum* |
| Clusiaceae | *Garcinia parvifolia* | Scan (6) |
| Compositae | *Mikania cordata* | Scan (9) |
| Connaraceae | *Agelaea borneensis* | *Ad libitum* |
| Cucurbitaceae | *Trichosanthes quinqualata* | *Ad libitum* |
| Dilleniaceae | *Dillenia borneensis* | Scan (1) |
|  | *Dillenia excelsa* | Scan (10) |
|  | *Tetracera scandens* | *Ad libitum* |
| Dipterocarpaceae | *Vatica venulosa* | Scan (4) |
| Ebenaceae | *Diospyros sp.* | Scan (2) |
|  | *Diospyros sp.1* | *Ad libitum* |
|  | *Diospyros tuberculata* | *Ad libitum* |
|  | *Diospyros walichii* | *Ad libitum* |
| Erythroxylaceae | *Erythroxylum cuneatum* | *Ad libitum* |
| Euphorbiaceae | *Mallotus floribundus* | Scan (6) |
|  | *Mallotus muticus* | Scan (28) |
| **Lamiaceae** | ***Vitex pinnata*** | **Scan** (**39)** |
| Lauraceae | *Actinodaphne glomerata* | *Ad libitum* |
|  | *Dehaasia sp.* | Scan (3) |
|  | *Litsea accedens* | Scan (1) |
| Lecythidaceae | *Planchonia valida* | Scan (1) |
| Leguminosae | *Caesalpinia sp.1* | Scan (2) |
|  | *Cynometra ramiflora* | Scan (5) |
|  | *Cynometra sp.* | *Ad libitum* |
|  | *Dalbergia stipulaceae* | Scan (1) |
|  | *Parkia cf. javanica* | *Ad libitum* |
| Lophopyxidaceae | *Lophopyxis maingayi* | Scan (4) |
| Lythraceae | *Duabanga moluccana* | Scan (1) |
|  | *Lagerstroemia speciosa* | *Ad libitum* |
| **Malvaceae** | *Colona serratifolia* | Scan (20) |
|  | *Kleinhovia hospita* | Scan (5) |
|  | *Pterospermum diversifolium* | Scan (3) |
|  | ***Pterospermum elongatum*** | **Scan** **(104)** |
|  | *Pterospermum sp.* | *Ad libitum* |
| **Moraceae** | *Ficus benjamina* | Scan (1) |
|  | *Ficus crassiramea* | Scan (11) |
|  | *Ficus fistulosa* | Scan (4) |
|  | ***Ficus racemosa*** | **Scan** **(848)** |
|  | *Ficus sp.1* | Scan (1) |
| Myrtaceae | *Syzygium cf. brachypodum* | *Ad libitum* |
|  | *Syzygium fastigiatum* | *Ad libitum* |
| Passifloraceae | *Passiflora foetida* | Scan (4) |
| Phyllanthaceae | *Antidesma thwaitesianum* | *Ad libitum* |
|  | *Bridelia penangiana* | *Ad libitum* |
|  | *Bridelia stipularis* | Scan (2) |
|  | *Cleistanthus obligonfolius* | *Ad libitum* |
|  | *Cleistanthus sp.* | Scan (15) |
|  | *Glochidion sp.* | *Ad libitum* |
| Putranjivaceae | *Drypetes sp.* | Scan (9) |
| Rhamnaceae | *Zizyphius borneensis* | *Ad libitum* |
| **Rubiaceae** | *Ludekia borneensis* | Scan (30) |
|  | *Mitragyna speciosa* | Scan (6) |
|  | ***Nauclea orientalis*** | **Scan** **(178)** |
|  | *Nauclea subdita* | Scan (2) |
| **Tetramelaceae** | ***Octomeles sumatrana*** | **Scan** **(195)** |
| Unknown | Unknown spp. | Scan (60) |
|  | Unknown sp.1 | Scan (5) |
|  | Unknown sp.2 | Scan (12) |
| Urticaceae | *Poikilospermum suaveolens* | Scan (1) |
| Vitaceae | *Cayratia trifolia* | Scan (11) |
|  | *Leea indica* | Scan (1) |

^i^ Numbers in brackets refer to the number of feeding occurrences recorded during scan sampling; the top-five consumed plants are in bold. Unknown spp. includes several undistinguishable plants, different from Unknown sp. 1 and 2., and is not included in the 67 food plant taxa.
