# Supplementary material for: Using DNA metabarcoding and direct behavioural observations to identify the diet of proboscis monkeys (Nasalis larvatus) in the Kinabatangan Floodplain, Sabah
Source: PLoS One. 2025 Jan 3;20(1):e0316752. doi: 10.1371/journal.pone.0316752 (PMC11698349; doi:10.1371/journal.pone.0316752)
Supplement: S2 Table — (DOCX) [file pone.0316752.s003.docx]

**SUPPORTING INFORMATION**

S2 Table. List of food plant taxa (n=100) recorded in proboscis monkey faeces using DNA metabarcoding

| **Family** | **MOTU** | **Nb of faeces** | **F_O_** | **Rank** | **Count of reads** |
| --- | --- | --- | --- | --- | --- |
| Anacardiaceae | **Dracontomelon dao** | **109** | **0.70** | **5** | **219027** |
|  | Anacardiaceae 1 | 19 | 0.12 | 46 | 19614 |
|  | Anacardiaceae 2 | 5 | 0.03 | 80 | 2293 |
| Annonaceae | Polyalthia 1 | 68 | 0.44 | 14 | 81578 |
|  | Annonaceae 1 | 2 | 0.01 | 86 | 1360 |
| Apocynaceae | Apocynaceae 1 | 55 | 0.35 | 17 | 47099 |
|  | Apocynaceae 2 | 6 | 0.04 | 76 | 13766 |
| Araceae | Pothos brevistylus | 30 | 0.19 | 32 | 5714 |
| Burseraceae | Canarium denticulatum | 38 | 0.25 | 27 | 49628 |
| Calophyllaceae | Mesua oblongifolia | 14 | 0.09 | 66 | 18848 |
| Capparaceae | Crateva religiosa | 1 | 0.01 | 96 | 1243 |
| Chrysobalanaceae | Chrysobalanaceae 1 | 20 | 0.13 | 42 | 6107 |
| Compositae | Mikania cordata | 38 | 0.25 | 28 | 5999 |
| Convolvulaceae | Erycibe grandifolia | 55 | 0.35 | 18 | 7093 |
|  | Merremia umbellata | 23 | 0.15 | 40 | 37394 |
| Cornaceae | Alangium javanicum | 15 | 0.10 | 63 | 10292 |
| Dilleniaceae | Dillenia 1 | 73 | 0.47 | 12 | 46686 |
| Dipterocarpaceae | Vatica 1 | 30 | 0.19 | 33 | 89704 |
| Ebenaceae | Diospyros 1 | 20 | 0.13 | 43 | 12314 |
| Elaeocarpaceae | Elaeocarpus 1 | 34 | 0.22 | 30 | 13632 |
|  | Elaeocarpaceae 1 | 15 | 0.10 | 64 | 8386 |
| Euphorbiaceae | **Mallotus 1** | **117** | **0.75** | **4** | **398420** |
|  | Euphorbiaceae 1 | 47 | 0.30 | 21 | 61929 |
|  | Mallotus floribundus | 43 | 0.28 | 23 | 19334 |
|  | Euphorbiaceae 2 | 33 | 0.21 | 31 | 6928 |
|  | Euphorbiaceae 3 | 18 | 0.12 | 50 | 60332 |
|  | Hancea 1 | 2 | 0.01 | 87 | 3269 |
|  | Mallotus 2 | 1 | 0.01 | 97 | 782 |
| Hypericaceae | Cratoxylum 1 | 6 | 0.04 | 77 | 3508 |
| Lamiaceae | Vitex 1 | 74 | 0.48 | 11 | 268104 |
|  | Teijsmanniodendron bogoriense | 16 | 0.10 | 57 | 14216 |
| Lauraceae | Lauraceae 1 | 49 | 0.32 | 20 | 25642 |
|  | Cryptocarya ferrea | 39 | 0.25 | 26 | 18026 |
| Lauraceae | Lauraceae 3 | 6 | 0.04 | 78 | 14782 |
| Lecythidaceae | Barringtonia pterita | 19 | 0.12 | 47 | 4396 |
|  | Planchonia valida | 9 | 0.06 | 72 | 9164 |
| Leguminosae | Leguminosae 1 | 75 | 0.48 | 10 | 129914 |
|  | Caesalpinia sp.1 | 64 | 0.41 | 15 | 46196 |
|  | Derris elegans | 61 | 0.39 | 16 | 42456 |
|  | Entada rheedii | 53 | 0.34 | 19 | 39678 |
|  | Leguminosae 2 | 29 | 0.19 | 35 | 9073 |
|  | Leguminosae 3 | 24 | 0.15 | 39 | 25776 |
|  | Leguminosae 4 | 18 | 0.12 | 51 | 2268 |
|  | Dalbergia stipulacea | 17 | 0.11 | 53 | 17210 |
|  | Leguminosae 8 | 16 | 0.10 | 58 | 87567 |
|  | Leguminosae 6 | 16 | 0.10 | 59 | 4148 |
|  | Leguminosae 7 | 16 | 0.10 | 60 | 2699 |
|  | Leguminosae 5 | 16 | 0.10 | 61 | 1843 |
|  | Leguminosae 10 | 11 | 0.07 | 70 | 5822 |
|  | Leguminosae 16 | 2 | 0.01 | 88 | 5241 |
| Lophopyxidaceae | Lophopyxis maingayi | 88 | 0.57 | 8 | 620673 |
|  | Lophopyxidaceae 4 | 4 | 0.03 | 84 | 3817 |
| Lythraceae | Lagerstroemia speciosa | 42 | 0.27 | 24 | 40077 |
|  | Lythraceae 1 | 5 | 0.03 | 81 | 9348 |
|  | Duabanga moluccana | 2 | 0.01 | 89 | 30164 |
| Malpighiaceae | Malpighiaceae 1 | 36 | 0.23 | 29 | 32783 |
| Malvaceae | Pterospermum 1 | 72 | 0.46 | 13 | 63054 |
|  | Microcos crassifolia | 29 | 0.19 | 36 | 3955 |
|  | Kleinhovia hospita | 2 | 0.01 | 90 | 9557 |
| Moraceae | **Ficus 1** | **149** | **0.96** | **2** | **528965** |
|  | Moraceae 1 | 29 | 0.19 | 37 | 13344 |
|  | Moraceae 2 | 18 | 0.12 | 52 | 1172 |
|  | Moraceae 3 | 8 | 0.05 | 73 | 14408 |
|  | Moraceae 4 | 7 | 0.05 | 75 | 5211 |
|  | Moraceae 5 | 5 | 0.03 | 82 | 9179 |
|  | Moraceae 8 | 1 | 0.01 | 98 | 345 |
| Myristicaceae | Myristicaceae 2 | 2 | 0.01 | 91 | 1778 |
| Myrtaceae | Syzygium 1 | 89 | 0.57 | 7 | 114577 |
|  | Syzygium 2 | 30 | 0.19 | 34 | 70160 |
|  | Myrtaceae 1 | 17 | 0.11 | 54 | 1414 |
|  | Myrtaceae 7 | 1 | 0.01 | 99 | 809 |
| Phyllanthaceae | **Bridelia 1** | **154** | **0.99** | **1** | **1992087** |
|  | Phyllanthaceae 1 | 27 | 0.17 | 38 | 1538 |
|  | Margaritaria indica | 20 | 0.13 | 44 | 30022 |
|  | Phyllanthaceae 2 | 17 | 0.11 | 55 | 25600 |
|  | Phyllanthaceae 3 | 15 | 0.10 | 65 | 661 |
|  | Bridelia 2 | 13 | 0.08 | 67 | 41442 |
|  | Phyllanthaceae 4 | 12 | 0.08 | 68 | 3654 |
|  | Phyllanthaceae 8 | 6 | 0.04 | 79 | 11404 |
|  | Phyllanthaceae 12 | 3 | 0.02 | 85 | 2123 |
| Polygalaceae | Xanthophyllum 1 | 21 | 0.14 | 41 | 2727 |
| Rhamnaceae | Ziziphus borneensis | 19 | 0.12 | 48 | 2084 |
| Rubiaceae | Rubiaceae 1 | 88 | 0.57 | 9 | 573173 |
|  | Neolamarckia cadamba | 20 | 0.13 | 45 | 130023 |
|  | Rubiaceae 2 | 16 | 0.10 | 62 | 1022 |
|  | Rubiaceae 6 | 5 | 0.03 | 83 | 10189 |
|  | Rubiaceae 13 | 2 | 0.01 | 92 | 4736 |
|  | Antirhea inaequalis | 2 | 0.01 | 93 | 343 |
|  | Uncaria 1 | 1 | 0.01 | 100 | 4416 |
| Salicaceae | Salicaceae 1 | 17 | 0.11 | 56 | 7745 |
| Sapindaceae | Dimocarpus longan | 19 | 0.12 | 49 | 32272 |
|  | Sapindaceae 1 | 10 | 0.06 | 71 | 6051 |
|  | Sapindaceae 2 | 2 | 0.01 | 94 | 3327 |
|  | Dimocarpus sp.1 | 2 | 0.01 | 95 | 451 |
| Simaroubaceae | Simaroubaceae 1 | 8 | 0.05 | 74 | 12420 |
| Symplocaceae | Symplocaceae 1 | 12 | 0.08 | 69 | 8854 |
| Tetramelaceae | **Octomeles sumatrana** | **122** | **0.79** | **3** | **649925** |
| Urticaceae | Poikilospermum suaveolens | 45 | 0.29 | 22 | 9437 |
| Vitaceae | Cayratia trifolia | 100 | 0.65 | 6 | 46868 |
|  | Tetrastigma lanceolarium | 41 | 0.26 | 25 | 13121 |

^i^ Rank numbers refer to the F_O_ score (i.e., Frequency of occurrences = the proportion of faecal samples containing the MOTU), and MOTUs with the top-five F_O_ score in bold.
