# Supplementary material for: Using DNA metabarcoding and direct behavioural observations to identify the diet of proboscis monkeys (Nasalis larvatus) in the Kinabatangan Floodplain, Sabah
Source: PLoS One. 2025 Jan 3;20(1):e0316752. doi: 10.1371/journal.pone.0316752 (PMC11698349; doi:10.1371/journal.pone.0316752)
Supplement: S3 Table — (DOCX) [file pone.0316752.s004.docx]

**SUPPORTING INFORMATION**

S3 Table. List of food plant families (n=39) recorded in proboscis monkey faeces

| **Familiy** | **MOTUs** | **Count reads** | **Nb fèces** | **F_o_** |
| --- | --- | --- | --- | --- |
| Phyllanthaceae | 9 | 2108531 | 154 | 0.99 |
| Moraceae | 7 | 572624 | 149 | 0.96 |
| Leguminosae | 14 | 419891 | 137 | 0.88 |
| Euphorbiaceae | 7 | 550994 | 128 | 0.83 |
| Tetramelaceae | 1 | 649925 | 122 | 0.79 |
| Anacardiaceae | 3 | 240934 | 115 | 0.74 |
| Vitaceae | 2 | 59989 | 114 | 0.74 |
| Myrtaceae | 4 | 186960 | 100 | 0.65 |
| Rubiaceae | 7 | 723902 | 94 | 0.61 |
| Lophopyxidaceae | 2 | 624490 | 92 | 0.59 |
| Malvaceae | 3 | 76566 | 88 | 0.57 |
| Lamiaceae | 2 | 282320 | 79 | 0.51 |
| Dilleniaceae | 1 | 46686 | 73 | 0.47 |
| Lauraceae | 3 | 58450 | 73 | 0.47 |
| Convolvulaceae | 2 | 44487 | 72 | 0.46 |
| Annonaceae | 2 | 82938 | 69 | 0.45 |
| Apocynaceae | 2 | 60865 | 55 | 0.35 |
| Lythraceae | 3 | 79589 | 49 | 0.32 |
| Urticaceae | 1 | 9437 | 45 | 0.29 |
| Elaeocarpaceae | 2 | 22018 | 43 | 0.28 |
| Burseraceae | 1 | 49628 | 38 | 0.25 |
| Compositae | 1 | 5999 | 38 | 0.25 |
| Malpighiaceae | 1 | 32783 | 36 | 0.23 |
| Araceae | 1 | 5714 | 30 | 0.19 |
| Dipterocarpaceae | 1 | 89704 | 30 | 0.19 |
| Sapindaceae | 4 | 42101 | 28 | 0.18 |
| Lecythidaceae | 2 | 13560 | 24 | 0.15 |
| Polygalaceae | 1 | 2727 | 21 | 0.14 |
| Chrysobalanaceae | 1 | 6107 | 20 | 0.13 |
| Ebenaceae | 1 | 12314 | 20 | 0.13 |
| Rhamnaceae | 1 | 2084 | 19 | 0.12 |
| Salicaceae | 1 | 7745 | 17 | 0.11 |
| Cornaceae | 1 | 10292 | 15 | 0.10 |
| Calophyllaceae | 1 | 18848 | 14 | 0.09 |
| Symplocaceae | 1 | 8854 | 12 | 0.08 |
| Simaroubaceae | 1 | 12420 | 8 | 0.05 |
| Hypericaceae | 1 | 3508 | 6 | 0.04 |
| Myristicaceae | 1 | 1778 | 2 | 0.01 |
| Capparaceae | 1 | 1243 | 1 | 0.01 |

^i^ Plant families are ranked by their frequency of occurrences (F_O_)
