# Supplementary material for: Using DNA metabarcoding and direct behavioural observations to identify the diet of proboscis monkeys (Nasalis larvatus) in the Kinabatangan Floodplain, Sabah
Source: PLoS One. 2025 Jan 3;20(1):e0316752. doi: 10.1371/journal.pone.0316752 (PMC11698349; doi:10.1371/journal.pone.0316752)
Supplement: S4 Table — (DOCX) [file pone.0316752.s005.docx]

**SUPPORTING INFORMATION**

S4 Table. List of the plant taxa (n=89) consumed by proboscis monkeys in the study site, combining both methods

| **Family** | **Genus** | **Taxa** | **DNA** | **DO** |
| --- | --- | --- | --- | --- |
| Anacardiaceae | *Buchanania* | *Buchanania arborescens* |  | 1 |
|  | *Dracontomelon* | *Dracontomelon dao* | 1 | 1 |
| Annonaceae | *Cananga* | *Cananga odorata* |  | 1 |
|  | *Polyalthia* | *Polyalthia obliqua* |  | 1 |
| Apocynaceae | *Alstonia* | *Alstonia iwahigensis* |  | 1 |
|  | *Rauvolfia* | *Rauvolfia sumatrana* |  | 1 |
| Araceae | *Pothos* | *Pothos brevistylus* | 1 | 1 |
| Burseraceae | *Canarium* | *Canarium denticulatum* | 1 |  |
| Calophyllaceae | *Mesua* | *Mesua oblongifolia* | 1 |  |
| Capparaceae | *Crateva* | *Crateva religiosa* | 1 |  |
| Chrysobalanaceae | Unknown 1 | Unknown 1 | 1 |  |
| Clusiaceae | *Garcinia* | *Garcinia parvifolia* |  | 1 |
| Compositae | *Mikania* | *Mikania cordata* | 1 | 1 |
| Connaraceae | *Agelaea* | *Agelaea borneensis* |  | 1 |
| Convolvulaceae | *Erycibe* | *Erycibe grandifolia* | 1 |  |
|  | *Merremia* | *Merremia umbellata* | 1 |  |
| Cornaceae | *Alangium* | *Alangium javanicum* | 1 |  |
| Cucurbitaceae | *Trichosanthes* | *Trichosanthes quinqualata* |  | 1 |
| Dilleniaceae | *Dillenia* | *Dillenia borneensis* |  | 1 |
|  | *Dillenia* | *Dillenia excelsa* |  | 1 |
|  | *Tetracera* | *Tetracera scandens* |  | 1 |
| Dipterocarpaceae | *Vatica* | *Vatica venulosa* |  | 1 |
| Ebenaceae | *Diospyros* | *Diospyros sp.1* |  | 1 |
|  | *Diospyros* | *Diospyros tuberculata* |  | 1 |
|  | *Diospyros* | *Diospyros walichii* |  | 1 |
| Elaeocarpaceae | *Elaeocarpus* | *Elaeocarpus 1* | 1 |  |
| Erythroxylaceae | *Erythroxylum* | *Erythroxylum cuneatum* |  | 1 |
| Euphorbiaceae | *Hancea* | *Hancea 1* | 1 |  |
|  | *Mallotus* | *Mallotus floribundus* | 1 | 1 |
|  | *Mallotus* | *Mallotus muticus* |  | 1 |
| Hypericaceae | *Cratoxylum* | *Cratoxylum 1* | 1 |  |
| Lamiaceae | *Teijsmanniodendron* | *Teijsmanniodendron bogoriense* | 1 |  |
|  | *Vitex* | *Vitex pinnata* |  | 1 |
| Lauraceae | *Actinodaphne* | *Actinodaphne glomerata* |  | 1 |
|  | *Cryptocarya* | *Cryptocarya ferrea* | 1 |  |
|  | *Dehaasia* | *Dehaasia sp.* |  | 1 |
|  | *Litsea* | *Litsea accedens* |  | 1 |
| Lecythidaceae | *Barringtonia* | *Barringtonia pterita* | 1 |  |
|  | *Planchonia* | *Planchonia valida* | 1 | 1 |
| Leguminosae | *Caesalpinia* | *Caesalpinia sp.1* | 1 | 1 |
|  | *Cynometra* | *Cynometra ramiflora* |  | 1 |
|  | *Dalbergia* | *Dalbergia stipulaceae* | 1 | 1 |
|  | *Derris* | *Derris elegans* | 1 |  |
|  | *Entada* | *Entada rheedii* | 1 |  |
|  | *Parkia* | *Parkia cf. javanica* |  | 1 |
| Lophopyxidaceae | *Lophopyxis* | *Lophopyxis maingayi* | 1 | 1 |
| Lythraceae | *Duabanga* | *Duabanga moluccana* | 1 | 1 |
|  | *Lagerstroemia* | *Lagerstroemia speciosa* | 1 | 1 |
| Malpighiaceae | Unknown 2 | Unknown 2 | 1 |  |
| Malvaceae | *Colona* | *Colona serratifolia* |  | 1 |
|  | *Kleinhovia* | *Kleinhovia hospita* | 1 | 1 |
|  | *Microcos* | *Microcos crassifolia* | 1 |  |
|  | *Pterospermum* | *Pterospermum diversifolium* |  | 1 |
|  | *Pterospermum* | *Pterospermum elongatum* |  | 1 |
| Moraceae | *Ficus* | *Ficus benjamina* |  | 1 |
|  | *Ficus* | *Ficus crassiramea* |  | 1 |
|  | *Ficus* | *Ficus fistulosa* |  | 1 |
|  | *Ficus* | *Ficus racemosa* |  | 1 |
|  | *Ficus* | *Ficus sp.1* |  | 1 |
| Myristicaceae | Unknown 3 | Unknown 3 | 1 |  |
| Myrtaceae | *Syzygium* | *Syzygium cf. brachypodum* |  | 1 |
|  | *Syzygium* | *Syzygium fastigiatum* |  | 1 |
| Passifloraceae | *Passiflora* | *Passiflora foetida* |  | 1 |
| Phyllanthaceae | *Antidesma* | *Antidesma thwaitesianum* |  | 1 |
|  | *Bridelia* | *Bridelia penangiana* |  | 1 |
|  | *Bridelia* | *Bridelia stipularis* |  | 1 |
|  | *Cleistanthus* | *Cleistanthus obligonfolius* |  | 1 |
|  | *Glochidion* | *Glochidion sp.* |  | 1 |
|  | *Margaritaria* | *Margaritaria indica* | 1 |  |
| Polygalaceae | *Xanthophyllum* | *Xanthophyllum 1* | 1 |  |
| Putranjivaceae | *Drypetes* | *Drypetes sp.* |  | 1 |
| Rhamnaceae | *Zizyphius* | *Zizyphius borneensis* | 1 | 1 |
| Rubiaceae | *Antirhea* | *Antirhea inaequalis* | 1 |  |
|  | *Ludekia* | *Ludekia borneensis* |  | 1 |
|  | *Mitragyna* | *Mitragyna speciosa* |  | 1 |
|  | *Nauclea* | *Nauclea orientalis* |  | 1 |
|  | *Nauclea* | *Nauclea subdita* |  | 1 |
|  | *Neolamarckia* | *Neolamarckia cadamba* | 1 |  |
|  | *Uncaria* | *Uncaria 1* | 1 |  |
| Salicaceae | Unknown 4 | Unknown 4 | 1 |  |
| Sapindaceae | *Dimocarpus* | *Dimocarpus longan* | 1 |  |
|  | *Dimocarpus* | *Dimocarpus sp.1* | 1 |  |
| Simaroubaceae | Unknown 5 | Unknown 5 | 1 |  |
| Symplocaceae | Unknown 6 | Unknown 6 | 1 |  |
| Tetramelaceae | *Octomeles* | *Octomeles sumatrana* | 1 | 1 |
| Urticaceae | *Poikilospermum* | *Poikilospermum suaveolens* | 1 | 1 |
| Vitaceae | *Cayratia* | *Cayratia trifolia* | 1 | 1 |
|  | *Leea* | *Leea indica* |  | 1 |
|  | *Tetrastigma* | *Tetrastigma lanceolarium* | 1 |  |

^i^ “DNA” refers to DNA metabarcoding method, “DO” to direct behavioural observation method, and “1” indicates that the taxa was detected by a method.
