# Supplementary material for: Using DNA metabarcoding and direct behavioural observations to identify the diet of proboscis monkeys (Nasalis larvatus) in the Kinabatangan Floodplain, Sabah
Source: PLoS One. 2025 Jan 3;20(1):e0316752. doi: 10.1371/journal.pone.0316752 (PMC11698349; doi:10.1371/journal.pone.0316752)
Supplement: Attachment — Submitted filename: Response to the academic editor secound round.docx [file pone.0316752.s008.docx]

**Response to the academic editor’s comments - PONE-D-24-28309 – second round**

**Authors' overall responses to the academic editor from PLoS ONE**

Thank you for your feedback on our manuscript. We have made the requested revision, focusing on ensuring compliance with licensing requirements for the figures. Below, we provide details of this change.

**We've checked your submission and before we can proceed, we need you to address the following issues:**

**1. Thank you for informing us that the map image is derived from previously published article (https://doi.org/10.1371/journal.pone.0174891). If your submission file inventory includes a map, we will ask you to provide a direct link to the source of the basemap and provide attribution to this source in the corresponding figure legend. We ask you to provide information regarding the terms of use or license information for the map (https://journals.plos.org/plosone/s/licenses-and-copyright#loc-licenses-for-data).**

**Please note that this is also a requirement of CCBY 4.0 licence (https://creativecommons.org/licenses/by/4.0/). As with all content, we ask that authors respect map providers’ requirements for attribution.**

The original Figure 1 was created some time before our manuscript submission to PLoS ONE. We were unable to retrieve the original shapefiles used for subpanel A. As a result, we rebuilt this subpanel using shapefiles that are compliant with the PLoS requirements. We established and edited (on Keynote) a map of Borneo using shapefiles from GADM (https://gadm.org).

As stated on their website: *“Using the data to create maps for publishing of academic research articles is allowed. Thus you can use the maps you made with GADM data for figures in articles published by PLOS, Springer Nature, Elsevier, MDPI, etc. You are allowed (but not required) to publish these articles (and the maps they contain) under an open license such as CC-BY as is the case with PLOS journals and may be the case with other open access articles*."

You can find more information about the license on the following webpage: https://gadm.org/license.html.

Subpanels B and C were drawn based on a figure available in a previously published article by one of the co-authors (Danica Stark, 2017) in PLoS ONE and are therefore licensed under CC BY 4.0.

The revised figure should no longer contain any elements that might raise licensing issues under PLoS ONE's requirements.

The updated Figure 1 is included in the new submission. We have also revised the figure caption to properly credit the data and map providers, ensuring full traceability. These modifications are highlighted in the "*Revised Manuscript with Track Changes.docx*" file.
